# The Potential Clinical Utility of Auditory P3b Amplitude for Clinical High Risk

**DOI:** 10.3389/fpsyg.2022.876092

**Published:** 2022-06-16

**Authors:** Xiaoli Liu, Haiyun Zhou, Changzhou Hu, Haihang Yu, Jucai Chu, Bifen Zhou

**Affiliations:** ^1^Ningbo Kangning Hospital, Ningbo, China; ^2^Lishui Second People's Hospital, Lishui, China; ^3^Taizhou Second People's Hospital, Taizhou, China

**Keywords:** auditory P300, clinical high risk (CHR), auditory oddball task, clinical outcomes, ERP

Individuals were found to be at clinical high risk (CHR) of conversion from prodromal syndrome to psychosis (Sentissi et al., 2017). A longitudinal cohort study reported CHR individuals were at high risk for transitioning to psychosis within the first 2 years (Nelson et al., 2013). A meta-analysis about future transition to psychosis, comprising a total of 2,502 patients, reported that the transitioning risk rate in CHR patients is 18% at 6 months of follow-up, 22% at 1 year, 29% at 2 years, 32% at 3 years, and 36% after 3 years (Fusar-Poli et al., 2012a). Patients with shorter duration of untreated psychosis had a better response to clinical interventions (Max et al., 2005; Rosengard et al., 2019). Early treatment aims to relieve psychotic prodromal symptoms and reduce the conversion risk to schizophrenia. Reflecting CHR clinical status helps psychiatrists set distinct treatment targets and develop an appropriate treatment program to remit the risk state. Consequently, finding objective indicators is important to improve the accuracy of CHR clinical identification and facilitate aggressive interventions.

Previous studies found that auditory P3b amplitude was associated with future conversion to psychosis and the time to this transition (van Tricht et al., 2010; Lepock et al., 2018; Graber et al., 2019). Furthermore, one study conducted by Hamilton was the largest longitudinal study with higher statistical power and confidence level to show that the auditory reduced p3b amplitude (evoked by infrequent targets and required response) not P3a (evoked by infrequent novel targets and required no response) was sensitive to the two clinical outcomes of conversion to psychosis and remission from CHR prodromal syndrome, but it could not differ CHR conversion and the CHR symptom persistence (Hamilton et al., 2019).

Hamilton et al.'s study inspires ideas for future study. Biological changes may underlie disorder onset and recurrence (Kennis et al., 2020). A reliable biomarker can be used in differential diagnosis and be measured and assessed as an indicator of pathogenic processes or pharmacological responses to therapies. The longitudinal study demonstrated a decreased P3b amplitude was reported in CHR patients and P3b amplitude is one of indicators that suggested a remission state from CHR. It is unknown whether the auditory P3b amplitude can be used as a dynamic and responsive indicator for pharmacological treatment. There is limited research focused on it. A decreased P300 amplitude was reported in mental diseases which suggested that P3b cannot be used in differentiating diagnosis. However, future studies exploring the reduced P3b amplitude of CHR with a trend toward normalization after pharmacological treatment are of great significance to improve P3b reliability and facilitate the P3b application in CHR clinical treatment.

Second, it inspired us to determine whether rehabilitation treatment targeted on improving the individual's P3b amplitude would then reduce the conversion risk. P3b reflects a top-down attentional processing, a mechanism that leads to cognitive categorization and context updating and possibly associates with prefrontal brain structures (Polich, 2007; Twomey et al., 2015; Rac-Lubashevsky and Kessler, 2019). Top-down attention refers to an internally guided attention process that is purely based on prior knowledge, willful plans, and current goals (Helfrich et al., 2019). Hence, we wondered if CHR individuals receiving rehabilitation training relevant to top-down attention exhibited a lower conversion risk than those that had not received attention rehabilitation. In addition, research about the neural mechanism of top-down attention supposed that higher cortical activations in the prefrontal cortex (PFC) and posterior parietal cortex (PPC) have been thought of as an important source of top-down attention (Katsuki and Constantinidis, 2014). Can increasing the P3b amplitude *via* modulating PFC or PPC activation improve the top-down attention process? Non-invasive brain stimulation (NIBS) such as transcranial direct current stimulation (tDCS) not only modulates cortical excitability and temporarily increases brain plasticity for the targeted cortex but also elicits effects on attention (Hallett, 2007; Polanía et al., 2018; Begemann and Brand, 2020). A future study could be designed to investigate the effect of tDCS treatment on P3b amplitude, thereby reducing the risk of CHR conversion.

Lastly, future studies can establish a well-performing risk calculator included P300 and other clinical information. Then the risk calculator can be used to predict the individualized probability of transition risk and better early identification and intervention. CHR patients have brain abnormalities at neuroanatomical (Fusar-Poli et al., 2012b), functional (Schmidt et al., 2013), and chemical levels (Allen et al., 2012). Combining neurocognitive, neuroimaging, or neurophysiological data with clinical information may further facilitate the better performance of the risk calculator. Cannon et al. developed a risk calculator prediction model with the data from NAPLS-2, which used demographic, clinical, neurocognitive, and psychosocial characteristics as predictors, and the positive predictive value can be increased by 60–80% (Cannon et al., 2016), but this calculator did not incorporate any electrophysiological or neuroimaging markers. An imaging-based risk model applying proton magnetic resonance spectroscopy showed high predictive values for prediction of conversion risk to CHR (Kegeles et al., 2020). Collin et al. observed that the prediction model-absorbed brain functional connectivity data had a better prediction effect for CHR patients (Collin et al., 2020). But these brain-imaging methods are very expensive and require high-load data processing. Compared with them, P300 evoked by the oddball task, as a low-cost routine medical electrophysiological examination, can be used to assess the progression of mental illness and to evaluate the efficacy of interventions, and has great potential in clinical implementation (especially in Chinese primary hospitals). Although P3b, as an event-related potential, records the sensor-level electrophysiological signals, the simplicity of the oddball task, the depth of understanding of P3b, and the suggestive previous literature combines to form a persuasive rationale for the potential clinical utility of P3b for CHR.

## Author Contributions

JC and BZ designed the study. XL and HZ performed the study and wrote the manuscript. CH and HY revised the manuscript. All authors have read and approved the final version of the manuscript.

## Funding

This study was supported by the Medical Science and Technology Project in Ningbo (2018A54, 2019Y23), the Ningbo Health Branding Subject Fund (PPXK2018-08), the Lishui Public Welfare Project (2021SJZC046), and the Zhejiang Medical and Health Science and Technology Plan Project (2019RC269).

## Conflict of Interest

The authors declare that the research was conducted in the absence of any commercial or financial relationships that could be construed as a potential conflict of interest.

## Publisher's Note

All claims expressed in this article are solely those of the authors and do not necessarily represent those of their affiliated organizations, or those of the publisher, the editors and the reviewers. Any product that may be evaluated in this article, or claim that may be made by its manufacturer, is not guaranteed or endorsed by the publisher.
